# Hospital length of stay and its predictors among surgical patients at public hospitals in Addis Ababa, Ethiopia

**DOI:** 10.3389/fsurg.2025.1431369

**Published:** 2025-07-22

**Authors:** Samuel Dessu Sifer, Abduselam Ahmed Abdela, Milkiyas Solomon Getachew, Redait Awoke Assefa, Abatalem Minlargeh Abere

**Affiliations:** ^1^ School of Public Health, Yekatit 12 Hospital Medical College, Addis Ababa, Ethiopia; ^2^ Department of Public Health, Yanet College, Addis Ababa, Ethiopia; ^3^ Department of Public Health, College of Medicine and Health Sciences, University of Gondar, Gondar, Ethiopia; ^4^ Department of Internal Medicine, College of Medicine and Health Sciences, University of Gondar, Gondar, Ethiopia; ^5^ Advance Medium Clinic, Jimma, Ethiopia

**Keywords:** length of hospital stay, predictors, surgical patients, public hospitals, Addis Ababa

## Abstract

**Background:**

Reducing the length of hospital stay can significantly lower healthcare costs, minimize the risk of hospital-acquired infections and complications, and improve patient well-being. Accordingly, this study aimed to assess the length of hospital stay and its predictors among surgical patients at public hospitals in Addis Ababa, Ethiopia.

**Methods:**

A prospective cohort study was conducted among 394 surgical patients from August 1 and November 30, 2023. The length of hospital stay was analyzed using Kaplan–Meier survival curves, and group differences were assessed with the log-rank test. Variables with a *p*-value less than 0.05 in the multivariable Cox proportional hazards model were considered statistically significant predictors.

**Result:**

The median length of hospital stay was 6 days (IQR 5, 6). Comorbidity (AHR: 3.32; 95% CI: 1.01, 10.85), infection (AHR: 3.14; 95% CI: 1.77, 12.72), weight loss (AHR: 1.62; 95% CI: 1.03, 2.56), change in dietary pattern (AHR: 1.52; 95% CI; 1.98, 2.35) and change in functional capacity (AHR: 1.53; 95% CI: 1.12, 2.13) were independent predictors of length of hospital stay.

**Conclusion and recommendations:**

Our findings indicate that prolonged hospitalization is often a consequence of delayed recovery from surgery, particularly among patients with complicating conditions such as comorbidities, infections, poor nutritional status, and limited functional capacity. These findings underscore the need for integrated preoperative assessments and targeted interventions to optimize patient conditions, enhance postoperative recovery, and reduce hospital stay duration in resource-limited settings.

## Background

Shortening the length of hospital stay (LOS) is a crucial metric for evaluating hospital management and the overall efficiency of the healthcare system (1). A reduced hospital stay not only has the potential to lower healthcare costs but also decreases the risk of infections and other hospital-acquired diseases, ultimately enhancing the quality of life for patients (1, 2). LOS also serves as a readily measurable outcome parameter, directly impacting hospital expenses and the economic implications of trauma and disease (2).

In contemporary healthcare, the length of a patient's hospital stay poses a significant challenge for hospital systems (2). Prolonged hospitalizations can disrupt patient flow and limit access to care, often resulting in bed shortages (3). As a result, reducing the length of stay (LOS) has become a critical priority, given its substantial impact on lowering healthcare costs (2, 3). Globally, various strategies have been implemented to address extended hospital stays, particularly those related to malnutrition. These interventions primarily aim to prevent and manage malnutrition within the hospital environment (4).

The duration of hospital stay is commonly used as a metric to evaluate the quality of care and the efficient use of healthcare resources (4). Prolonged length of stay (PLOS) is associated with increased utilization of hospital resources, a higher risk of hospital-acquired infections (HAIs), elevated complication and mortality rates, delays in timely treatment for critically ill patients, and exacerbation of hospital capacity shortages (2, 5). However, relying solely on PLOS as an indicator of surgical care quality has limitations (6). To more accurately assess the efficiency and quality of surgical care, additional parameters such as postoperative complications, HAIs, and mortality must also be considered (2, 5).

This is particularly crucial in low- and middle-income countries (LMICs), where the burden of major surgical procedures is high and the associated rates of complications and mortality remain substantial, making surgical safety a global health priority (7). Several factors—including patient age, comorbidities, type of surgery, initial condition upon admission, and length of hospital stay—can significantly influence surgical patient survival (8, 9). Furthermore, structural limitations in healthcare systems, such as the lack of essential and specialized resources in intensive care units (ICUs), contribute to poor outcomes in low-resource settings (10). Surgical patients admitted to general wards often present with diverse conditions and varying levels of severity, further complicating care and outcomes (9, 10). Existing studies on predictors of surgical mortality have been predominantly conducted in high-income countries, using advanced clinical and laboratory indicators and sufficient staffing—resources that are often scarce in low-income settings, where adverse outcomes are more frequent despite the heavier disease burden (10). Accordingly, this study aimed to determine the length of hospital stay and its nutritional predictors among surgical patients in public hospitals in Addis Ababa, Ethiopia.

## Methods and materials

### Study design, area and period

A prospective cohort study was conducted in the surgical wards of public hospitals in Addis Ababa, Ethiopia, from September 1 to November 30, 2023. The study was carried out at four selected hospitals: Ras Desta Memorial Hospital, Menelik II Comprehensive Specialized Hospital, Zewditu Memorial Hospital, and Yekatit 12 Hospital Medical College (11).

Follow-up began immediately after the surgical procedure and continued until the patient achieved recovery. The follow-up was terminated if the participant died, withdrew from the study, or was discharged following recovery. In this study, surgical patients who discontinued treatment or were transferred to another facility were operationally defined as “censored.”

### Population

The source population for this study comprised all patients admitted to surgical wards at Public Hospitals in Addis Ababa. The study population specifically included patients admitted to the surgical wards at Ras Desta Memorial Hospital, Menilik II Comprehensive Specialized Hospital, Zewditu Memorial Hospital, and Yekatit 12 Hospital Medical College. Patients admitted to surgical wards at Public Hospitals in Addis Ababa were eligible for inclusion, while clinically unstable or unconscious patients, comatose individuals, bedridden patients, obstetrics patients, those with dementia, emergency surgery cases, and patients undergoing chemo/radiotherapy were excluded from the study.

### Sample size determination and procedure

The sample size for the study was determined using a double population proportion formula with the following assumptions: 95% confidence interval, power of 80%, Z (standard normal distribution) with a confidence interval of 95% (*α* = 0.05), P1 (Proportion of prolonged length of hospital stay among malnourished patients) at 57.3% (12), P2 (Proportion of prolonged length of hospital stay among well-nourished) at 27.7%, pooled proportion at 20%, ratio (*r*) of 1, design effect (B) of 20%, allowable error (A) of 0.05, and considering a 10% non-response rate. This yielded a sample size of 398. The study participants were then selected at each hospital using a systematic sampling method.

### Variable

The dependent variable in this study was the time to recovery, while the independent variables included socio-demographic factors such as age, sex, religion, marital status, and occupation. Anthropometric variables like usual normal weight and present weight, along with clinical data including diagnosis at admission, type of surgery, changes in dietary intake, significant gastrointestinal symptoms, functional capacity, loss of subcutaneous fat, muscle wastage, edema, and ascites were also considered.

### Operational definition

Event (outcome): in this study, the event was patient who discharged from hospital on any particular day as a live (Recovered).

Length of hospital stay: LOS will be determined from the date of hospital admission to discharge/days from hospital admission to discharge.

Censored: Patients who died during hospitalization or stayed in the ward more than 30 days were treated as censored observations.

Weight loss was measured objectively by comparing patients' weight at admission with their weight at discharge. Clinically significant weight loss was defined as a reduction of ≥5% of admission weight during the hospital stay.

### Data collection procedure and data quality control measures

Data collection for this study involved the use of a structured checklist specifically designed for the follow-up study. Data collectors underwent one day of training to familiarize themselves with the data collection process. The data collection tool was initially prepared in English, translated into Amharic, and then translated back into English to ensure consistency. The data collection process included interviews and subsequent follow-ups, as well as physical examinations. Information was extracted from patient charts, and daily follow-ups were conducted to collect individual data.

A pre-test was conducted on 5% of the samples outside the study area one week before the actual data collection to ensure the effectiveness and appropriateness of the data collection tool. On-site, the completeness and consistency of the collected data were monitored, and questionnaires with missing variables were returned to data collectors for correction through revisits. Regular testing of measuring equipment, such as scales, was conducted during data collection. Faulty equipment was promptly replaced. Daily checks included testing each scale with a standard weight of at least 5 kg to maintain accuracy and reliability.

### Data processing and analysis

The data were manually cleaned and initially entered into Epi-data version 4.6. Subsequently, it was checked and cleaned for consistency and any missing values. The cleaned data were then exported to SPSS Version 25 for further analysis. Principal Component Analysis was employed to construct a wealth index based on household data, including ownership of fixed assets such as the type of house and its building materials, agricultural land ownership, animal ownership, source of drinking water, ownership and type of toilet facility, presence of a domestic servant, and having a savings account.

Descriptive analysis was conducted to describe the study variables, and frequency analysis was run for socio-demographic, economic, and disease classification. The prevalence of malnutrition based on Subjective Global Assessment (SGA) criteria was estimated. Kaplan–Meier survival curve analysis was performed. Both bivariate and multivariable Cox proportional hazard models were fitted to determine the predictors of length of hospital stay. Variables with a *p*-value less than 0.25 in bivariate analysis were considered as candidates for multivariable analysis, and variables with a *p*-value less than 0.05 in the multivariable Cox proportional hazard model were declared statistically significant. The backward stepwise regression model was utilized. Finally, the findings were presented using text, tables, and figures.

### Ethics approval and consent to participate

Ethical clearance for the study was obtained from the Institutional Review Board (IRB) of Yanet College. Verbal consent was obtained from each study participant before the commencement of data collection.

## Results

### Socio demographic characteristics

The study involved a total of 394 participants, achieving a response rate of 98.99%. The participants ranged in age from 18 to 42 years, with a mean age of 28.7 ± 5.64 years. More than half of the participants (52.5%) were below the mean age. The majority of the respondents were male (90.1%), and around one-fourth (25.9%) identified as housewives. Additionally, nearly one-tenth (9.9%) of the participants were from rural areas.

A proxy measure for socioeconomic status, the wealth index, was derived from household ownership of durable goods and housing characteristics. Principal Components Analysis (PCA) was employed to calculate the wealth index. The variables included in the PCA encompassed household assets (such as a watch, radio, television, mobile telephone, landline telephone, refrigerator, vehicle, etc.), access to utilities and infrastructure (e.g., electricity, type of cooking fuel used), livestock ownership (number of chickens, cattle, donkeys, etc.), crop production, monthly income, and possession of agricultural land in hectares. The computation revealed that 165 (41.9%), 149 (37.8%), and 80 (20.3%) of the participants were categorized as having low (poor), middle (medium), and high (rich) wealth status, respectively (Table 1).

**Table 1:** Socio demographic characteristics among surgical patients at public hospitals in Addis Ababa, Ethiopia, 2023.

Variables	Category	Frequency (*n*)	Percentage (%)
Age (in years)	Below mean	207	52.5
	Above mean	187	47.5
Sex	Male	355	90.1
	Female	39	9.9
Educational status	Unable to read and write	170	43.1
	Only read and write	105	26.6
	Primary	92	23.4
	Secondary	19	4.8
	College and above	8	2.0
Marital status	Single	255	64.7
	Married	131	33.2
	Divorced	5	1.5
	Widowed	2	0.5
Occupational status	Housewife	102	25.9
	Farmer	11	2.8
	Daily laborer	61	15.5
	Merchant	99	25.1
	Student	34	8.6
	Private employees	54	13.7
	Government employee	33	8.4
Living arrangement	Living alone	47	110.9
	With partners	222	56.3
	With parents	125	31.7
Wealth index	Lower	165	41.9
	Middle	149	37.8
	High	80	20.3

### Medical related factors

**Figure 1:**
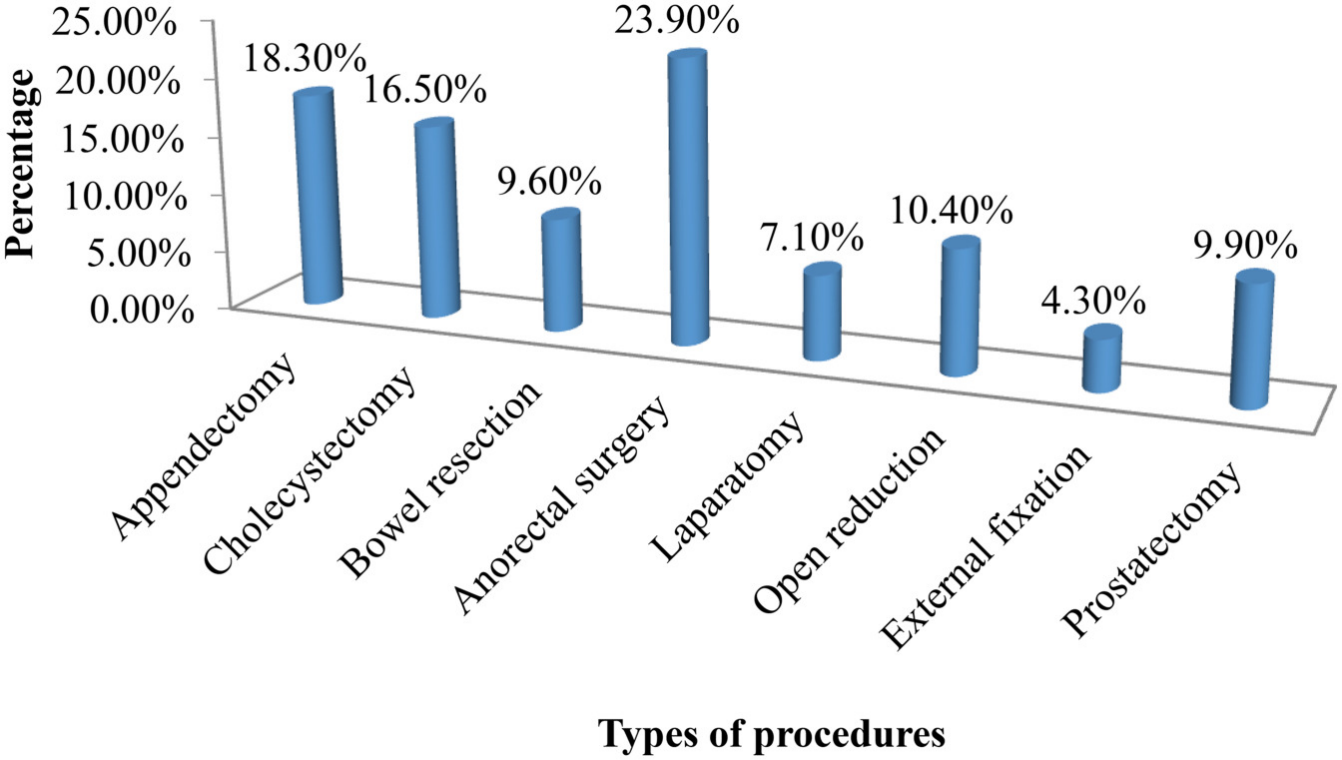
Type of surgical procedures conducted for patients at public hospitals table 2 in Addis Ababa, Ethiopia, 2023.

The distribution of admission diagnoses among the respondents revealed that the majority, 269 (68.3%), were admitted with gastrointestinal system problems, while the minimum. The remaining, 28 (7.1%), 58 (14.7%), and 39 (9.9%) were admitted with trauma, musculoskeletal system, and genitourinary system problems, respectively. Among the respondents, 25 (6.3%) had comorbid health problems, and 15 (3.8%) were dealing with current infections. Regarding current medication intake, 359 (91.1%) of the respondents were on drugs. Additionally, 9.9% had a history of past admission, and 3.3% had a history of past surgery (Figure 1).

### Nutritional related factors

Among the participants in the study, 55 (14.0%) reported experiencing weight loss. Similarly, nearly one-fifth (19.5%) reported a change in dietary intake. Among those with a change in dietary intake, 51 (66.2%) experienced borderline changes, and 24 (31.2%) had poor or decreasing changes. Regarding changes in functional capacity, 132 (33.5%) of the respondents reported a change. Among those with a change in functional capacity, 120 (90.9%) were working in suboptimal conditions.

### Time to recovery from surgery

**Figure 2:**
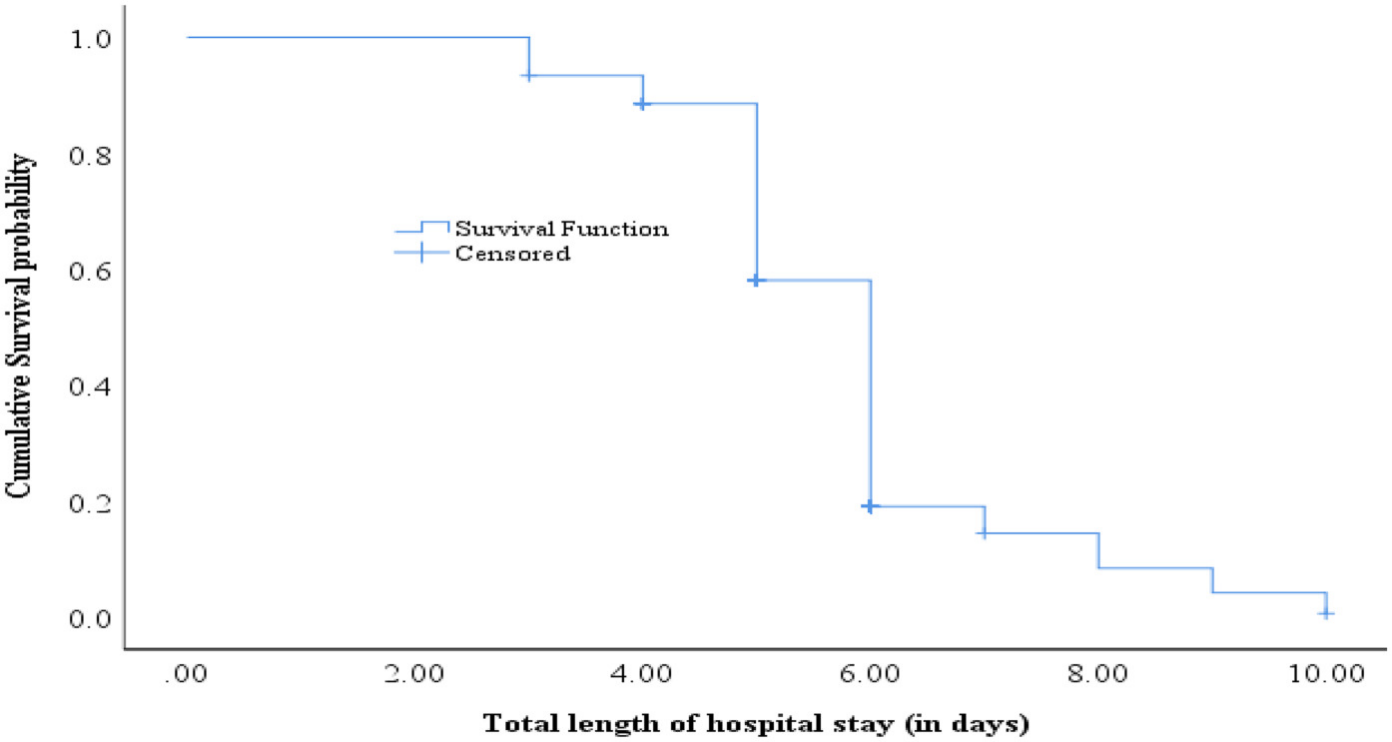
Survival time to the time of recovery from surgery among surgical a patients at public hospitals in Addis Ababa, Ethiopia, 2023.

The cumulative proportion of survival at the end of the third day was 93.4%. Additionally, it was 88.6%, 58.1%, 19.1%, and 14.4% at the end of the fourth, fifth, sixth, and seventh days, respectively. Similarly, it was 8.4%, 4.2%, and 0.6% at the end of the eighth, ninth, and tenth days, respectively. The median survival time was 6.0 (95% CI: 5.89, 6.11) (Figure 2).

### Length of hospital stay

**Figure 3:**
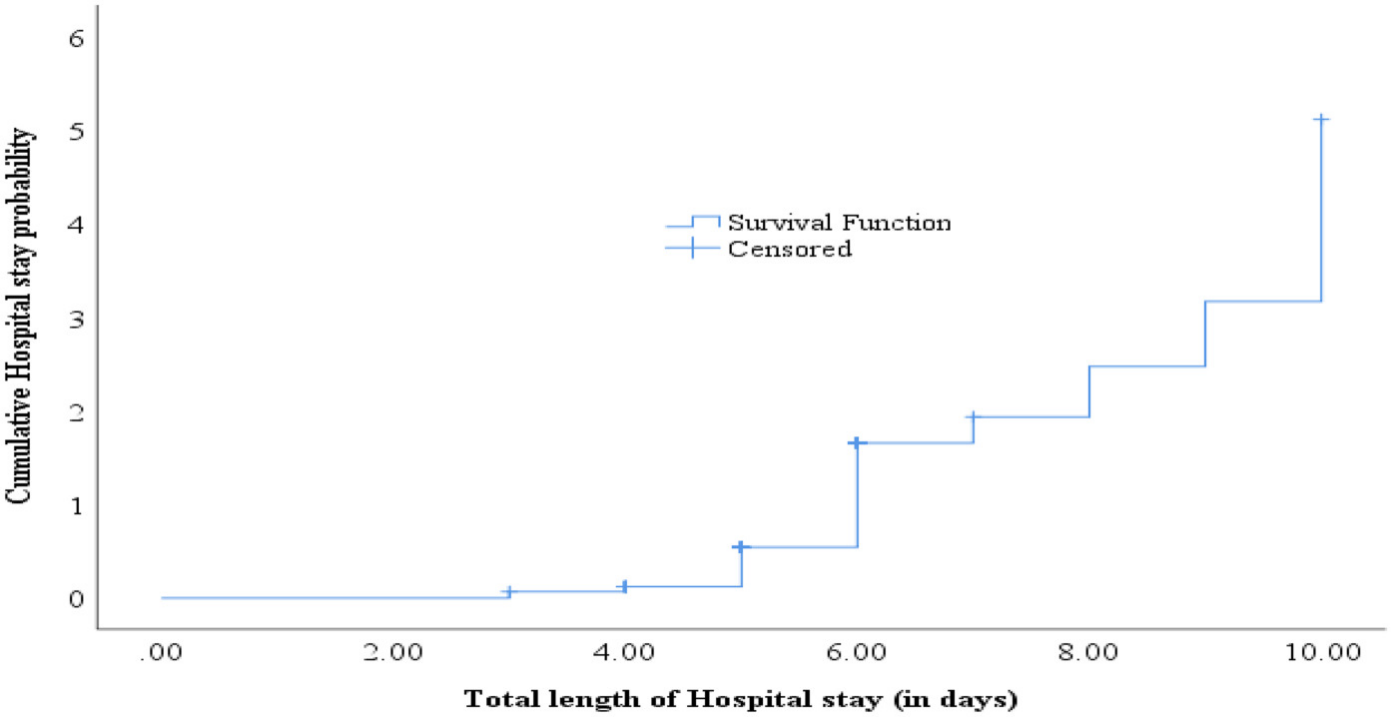
Length of hospital stay among surgical patients at public hospitals in Addis Ababa, Ethiopia, 2023.

The median length of hospital stay was 6 days (IQR 5, 6). Two hundred one (51.0%) had a hospital stay in the upper quartile (6 days or longer). Among the surgical patients, an equal number of 27 (6.9%) respondents stayed for three days and four days within the hospital. Similarly, 139 (35.3%) of the respondents stayed for five days, and 168 (42.6%) stayed for six days. In addition, 9 (2.3%), 10 (2.5%), 7 (1.8%), and 7 (1.8%) of surgical patients stayed for seven days, eight days, nine days, and ten days, respectively (Figure 3).

### Predictors of length of hospital stay from surgery

In this study, educational status, occupational status, admission primary diagnosis, comorbidity, having an infection, past admission history, weight loss, change in dietary pattern, change in functional capacity, loss of subcutaneous tissue, and muscle wasting were candidate variables for the multivariable Cox proportional hazard model. Among these candidate variables, comorbidity, infection, weight loss, change in dietary pattern, and change in functional capacity were identified as statistically significant predictors of the time to recovery from surgery.

Patients without comorbid health problems experienced approximately three times faster recovery after surgery compared to those with comorbidities (AHR: 3.32; 95% CI: 1.01, 10.85). Recovery from surgery was three times faster among patients without an infection compared to those with an infection (AHR: 3.14; 95% CI: 1.77, 12.72). Patients without weight loss had a 1.62 times faster recovery from surgery compared to those with weight loss (AHR: 1.62; 95% CI: 1.03, 2.56). Similarly, recovery from surgery was 1.52 times faster among patients with no change in dietary pattern compared to those with a change in dietary pattern (AHR: 1.52; 95% CI; 1.98, 2.35). Additionally, recovery from surgery among surgical patients with no change in functional capacity was 1.53 times faster compared to those with a change in functional capacity (AHR: 1.53; 95% CI: 1.12, 2.13) (Table 2).

**Table 2:** Predictors of time to recovery from surgery among patients at public hospitals in Addis Ababa, Ethiopia, 2023.

Variables	Category	Status		CHR (95% CI)	AHR (95% CI)
		Recovered	Censored		
Educational status	Unable to read and write	141	29	1	1
	Only read and write	91	14	0.92 (0.71, 1.19)	0.97 (0.73, 1.29)
	Primary	82	10	0.98 (0.75, 1.29)	0.94 (0.71, 1.24)
	Secondary	11	8	0.94 (0.51, 1.74)	1.24 (0.55, 2.78)
	College and above	3	5	0.37 (0.12, 1.16)*	0.57 (0.17, 1.86)
Occupational status	Housewife	72	30	1	1
	Farmer	9	2	1.52 (0.76, 3.03)*	0.91 (0.35, 2.39)
	Daily laborer	54	7	1.18 (0.83, 1.68)	0.73 (0.48, 1.09)
	Merchant	93	6	1.23 (0.91, 1.68)*	0.78 (0.53, 1.16)
	Student	24	10	0.86 (0.54, 1.38)	0.72 (0.43, 1.20)
	Private employee	50	4	1.14 (0.79, 1.64)	0.64 (0.41 (1.01)
	Government employee	26	7	1.13 (0.72, 1.77)	0.73 (0.44, 1.19)
Comorbidity	Yes	3	22	1	1
	No	325	44	6.92 (2.22, 21.58)*	3.32 (1.01, 10.85)**
Infection	Yes	3	12	1	1
	No	325	54	4.58 (1.47, 14.28)*	3.14 (1.77, 12.72)**
Past admission	Yes	13	26	1	1
	No	315	40	2.59 (1.49, 4.51)*	0.48 (0.19, 1.15)
Weight loss	Yes	23	32	1	1
	No	305	34	2.18 (1.43, 3.33)*	1.62 (1.03, 2.56)**
Change in dietary pattern	Yes	34	43	1	1
	No	294	23	2.02 (1.41, 2.88)*	1.52 (1.98, 2.35)**
Change in functional capacity	Yes	77	55	1	1
	No	251	11	1.69 (1.31, 2.16)*	1.53 (1.12, 2.13)**
Loss of subcutaneous tissue	None	237	46	1.85 (0.69, 4.99)*	0.87 (0.40, 1.88)
	Low to moderate	87	18	2.08 (0.76, 5.69)*	0.71 (0.25, 1.96)
	Sever	4	2	1	1
Muscle wasting	None			1.84 (0.68, 4.95)*	1.37 (0.62, 3.04)
	Low to moderate			2.17 (0.79, 5.97)*	1.43(0.71, 4.01
	Sever			1	1

*Indicates variables which have *p*-value <0.25 in bivariate analysis and ** indicates variables which have *p*-value <0.05 in multivariable analysis.

## Discussion

This study investigates the duration of hospital stay and its determinants among surgical patients in public hospitals in Addis Ababa, Ethiopia. Prolonged hospital stays can lead to increased healthcare costs, greater risk of complications, and higher rates of hospital-acquired infections, reinforcing the need for evidence-based interventions to minimize avoidable delays in surgical care. It is also associated with a shortened recovery period after surgery.

The cumulative proportion of patients still in the hospital at the end of the third day was 93.4%. Subsequently, this proportion decreased to 88.6%, 58.1%, 19.1%, and 14.4% by the end of the fourth, fifth, sixth, and seventh days, respectively. Furthermore, it dropped to 8.4%, 4.2%, and 0.6% by the end of the eighth, ninth, and tenth days, respectively.

The prolonged hospital stay observed in this study may be linked to the compromised nutritional status of patients, as poor nutrition has been previously associated with extended stays and treatment (13). Prolonged hospital stays could serve as a significant indicator of nutritional risk or status, reflecting the severity and impact of the disease, genetic factors, treatment duration, quality of care, and especially the adverse effects of malnutrition, such as impaired wound healing, reduced functional status, diminished quality of life, and increased hospital costs (14, 15). Additionally, it is noteworthy that malnourished individuals are commonly among those experiencing prolonged hospital stays (15, 16, 17).

The presence of comorbid health problems emerged as a predictor of prolonged hospital stay among surgical patients. Patients without comorbid health problems experienced approximately three times faster recovery after surgery compared to those with comorbidities. Our findings support the notion that the absence of comorbidities, infections, and nutritional problems significantly contributes to shorter hospital stays, which is consistent with global evidence emphasizing the importance of addressing these factors to enhance surgical recovery and reduce healthcare burdens (18, 19, 20).

Having an infection was identified as another predictor of time to recovery from surgery among patients undergoing surgery. In line with a study conducted in China (21), which found that having an infection increased the length of stay by 10.4 days, this study revealed that recovery from surgery is three times faster among patients with no infection compared to those with an infection. This association may be attributed to the complications related to infections, as well as the impact of medication intake during hospitalization. Additionally, both prolonged hospital stays and the presence of infections contribute to increased healthcare costs, straining medical resources and potentially exacerbating patient suffering and leading to medical disputes (22).

Among the nutrition-related factors, weight loss, change in dietary pattern, and change in functional capacity were identified as statistically significant predictors of time to recovery from surgery. Surgical patients who experienced no weight loss had 1.62 times faster recovery from surgery compared to those who had weight loss. This association may be linked to increased medical complications associated with weight loss (23). Additionally, it could be related to underlying diseases, catabolic stress resulting from surgical interventions, insufficient oral intake or fasting, as well as inadequate management of nutritional issues in patients (24). The study also revealed that compared to patients with a length of stay of at least three days, those who died in the hospital were more likely to have compromised nutritional status, experience unintentional weight loss, and have more severe diseases, including malignant neoplasms and a greater number of comorbidities (24).

Change in dietary pattern was identified as an independent predictor of time to recovery among surgical patients. Recovery from surgery was 1.52 times faster among patients who experienced no change in dietary pattern compared to those who had a change in dietary pattern. This association might be related to the fact that a change in dietary pattern increases the risk of infection, pressure ulcers, slows wound healing, reduces nutrient intestinal absorption, alters thermoregulation, and affects renal function. All these factors can contribute to a prolonged length of stay among patients (25).

Change in functional capacity of surgical patients was identified as one of the predictors of length of hospital stay. The study revealed that recovery from surgery among surgical patients who experienced no change in functional capacity was 1.53 times faster compared to those who had a change in functional capacity. This association could be attributed to increased muscle fatigue and reduced function, leading to complications such as infection (26). In addition, these factors may increase susceptibility to postoperative complications, such as pulmonary infections (e.g., pneumonia) or surgical site infections, due to weakened immune responses, poor circulation, and delayed tissue healing (27). The limitation of the study was, it determines the nutritional status using subjective global assessment tool, which may not be consistent with the laboratory investigations for the determination of the nutritional status.

Our findings should also be interpreted in light of global efforts to reduce hospital stay through Enhanced Recovery After Surgery (ERAS) protocols. The limited adoption of such protocols in the study settings may partly explain the observed length of stay, and their implementation could be considered as a strategy to improve surgical outcomes and resource utilization.

## Conclusion

Our findings indicate that prolonged hospitalization is often a consequence of delayed recovery from surgery, particularly among patients with complicating conditions such as comorbidities, infections, poor nutritional status, and limited functional capacity. These findings underscore the need for integrated preoperative assessments and targeted interventions to optimize patient conditions, enhance postoperative recovery, and reduce hospital stay duration in resource-limited settings.

## Data Availability

The original contributions presented in the study are included in the article/Supplementary Material, further inquiries can be directed to the corresponding author.
